# Improving the efficiency of integrated cancer screening delivery across multiple cancers: case studies from Idaho, Rhode Island, and Nebraska

**DOI:** 10.1186/s43058-022-00381-4

**Published:** 2022-12-16

**Authors:** Florence K. L. Tangka, Sujha Subramanian, Sonja Hoover, Charlene Cariou, Becky Creighton, Libby Hobbs, Amanda Marzano, Andrea Marcotte, Deirdre Denning Norton, Patricia Kelly-Flis, Melissa Leypoldt, Teri Larkins, Michelle Poole, Jennifer Boehm

**Affiliations:** 1 Centers for Disease Control and Prevention, 4770 Buford Highway, NE, Mailstop S107-4, Atlanta, GA 30341-3717 USA; 2 RTI International, 307 Waverley Oaks Road, Suite 101, Waltham, MA 02452-8413 USA; 3 Southwest District Health, 13307 Miami Lane, Caldwell, ID 83607 USA; 4 Idaho Comprehensive Cancer Control Program, Division of Public Health, Idaho Department of Health and Welfare, 450 W State Street, Boise, ID 83702 USA; 5 Bureau of Community and Environmental Health, Division of Public Health, Idaho Department of Health and Welfare, 450 W State Street, Boise, ID 83702 USA; 6 WellOne Primary Medical and Dental Care, 35 Village Plaza Way, North Scituate, RI 02857 USA; 7 Women’s and Men’s Health Programs, Lifespan Health Unit, Public Health, Nebraska Department of Health and Human Services, 301 Centennial Mall S, Lincoln, NE 68508 USA

**Keywords:** Integrated cancer screening, Breast cancer screening, Cervical cancer screening, Colorectal cancer screening, Patient navigator/navigation, Idaho, Rhode Island, Nebraska

## Abstract

**Background:**

Three current and former awardees of the Centers for Disease Control and Prevention’s Colorectal Cancer Control Program launched integrated cancer screening strategies to better coordinate multiple cancer screenings (e.g., breast, cervical, colorectal). By integrating the strategies, efficiencies of administration and provision of screenings can be increased and costs can be reduced. This paper shares findings from these strategies and describes their effects.

**Methods:**

The Idaho Department of Health and Welfare developed a Baseline Assessment Checklist for six health systems to assess the current state of policies regarding cancer screening. We analyzed the checklist and reported the percentage of checklist components completed. In Rhode Island, we collaborated with a nurse-patient navigator, who promoted cancer screening, to collect details on patient navigation activities and program costs. We then described the program and reported total costs and cost per activity. In Nebraska, we described the experience of the state in administering an integrated contracts payment model across colorectal, breast, and cervical cancer screening and reported cost per person screened. Across all awardees, we interviewed key stakeholders.

**Results:**

In Idaho, results from the checklist offered guidance on areas for enhancement before integrated cancer screening strategies, but identified challenges, including lack of capacity, limited staff availability, and staff turnover. In Rhode Island, 76.1% of 1023 patient navigation activities were for colorectal cancer screening only, with a much smaller proportion devoted to breast and cervical cancer screening. Although the patient navigator found the discussions around multiple cancer screening efficient, patients were not always willing to discuss all cancer screenings. Nebraska changed its payment system from fee-for-service to fixed cost subawards with its local health departments, which integrated cancer screening funding. Screening uptake improved for breast and cervical cancer but was mixed for colorectal cancer screening.

**Conclusions:**

The results from the case studies show that there are barriers and facilitators to integrating approaches to increasing cancer screening among primary care facilities. However, more research could further elucidate the viability and practicality of integrated cancer screening programs.

**Supplementary Information:**

The online version contains supplementary material available at 10.1186/s43058-022-00381-4.

Contributions to the literature

- Describes approaches used by three programs to plan, implement, and sustain integrated screening strategies to increase cancer screenings focused on cohorts of populations who are low-income and are medically underserved.
- Details facilitators and barriers to implementing integrated screening strategies from the perspective of the three programs.
- Provides cost and effectiveness measures for two programs.

## Background

The Centers for Disease Control and Prevention (CDC) has administered cancer control programs for nearly 30 years, since the establishment of the National Breast and Cervical Cancer Early Detection Program (NBCCEDP) in 1991 [1]. The NBCCEDP is funded by the CDC and focuses on providing mammograms and Pap tests to low-income, uninsured, and underinsured women nationally. In 2005, the CDC funded five awardees as part of the Colorectal Cancer Screening Demonstration Program to assess the feasibility of promoting and providing colorectal cancer screenings [2]. In 2009, the CDC expanded the program with the creation of the Colorectal Cancer Control Program (CRCCP) to promote and provide colorectal cancer screening and some diagnostic services. The CRCCP focused on low-income, uninsured, or underinsured men and women aged 50–64 years. Later, in 2015, the CRCCP shifted its focus to the promotion of colorectal cancer screening through the implementation of evidence-based interventions (e.g., provider assessment and feedback, provider and patient reminders, reduction of structural barriers) in primary care settings, as described in the *Guide to Community Preventive Services (Community Guide)* [3]*.*

In this paper, we evaluated integrated cancer screening strategies. We defined integrated cancer screening strategies as those that improve the coordination of multiple cancer screenings, increase efficiency in administering and providing the screenings, and potentially reduce cost. In addition, we defined integrated cancer screening programs as those that concurrently promote several cancer screenings (e.g., breast, cervical, colorectal). Examples include having a dedicated patient navigator to assist with multiple cancer screenings instead of focusing on one type of cancer, and using a streamlined contract system to integrate multiple funding sources at the state level to disburse as one funding stream instead of multiple streams to health systems.

We focused on one former and two current CRCCP programs that were at different phases during the study period: planning, implementation, and sustainment. The two current CRCCP programs were Idaho Department of Health & Welfare (ID-DHW) in the planning phase, and the Rhode Island Department of Health (RI-DOH) in the implementation phase. The Nebraska Department of Health & Human Services (NE-DHHS), a former CRCCP awardee during this study period, was in its sustainment phase using state funding, not federal funding. ID-DHW evaluated optimal approaches (e.g., provider assessment and feedback, patient reminders) implemented by its health system partners to deliver integrated cancer screening strategies to promote screening for colorectal, breast, and cervical cancers. RI-DOH partnered with a federally qualified health center (FQHC) to implement patient navigation services, mainly for colorectal cancer screening promotion, but included breast and cervical cancer screening promotion when appropriate. NE-DHHS integrated cancer screening through its payment model.

We conducted case studies of the three programs to identify approaches used to plan, implement, and sustain integrated delivery of strategies for cancer screenings that focused on cohorts of populations who have low income and are medically underserved. We described facilitators and barriers to implementing integrated screening strategies from the perspective of the three programs and provided cost and effectiveness measures for two programs. Lessons learned from these programs can guide future implementation efforts to jointly promote and efficiently increase cancer screenings at FQHCs and among other health care delivery systems in communities with limited resources.

## Methods

In this section, we describe for each program the components we analyzed (e.g., program planning across multiple health systems, implementation of a patient navigation program, sustainment of a statewide payment system), how we analyzed them, the data we collected, and the analyses completed. A summary of the three programs is shown in Table 1.

**Table 1:** Summary of phase, program description, and integration model evaluated by three colorectal cancer control programs

Phase	Program description	Integration approach or model evaluated
Planning	Idaho Department of Health & Welfare worked with 6 health system partners^a^ that implemented evidence-based interventions to promote colorectal cancer screening and, in many instances, to promote breast and cervical cancer screenings, as well.	Conducting baseline assessment to understand optimal approaches (e.g., provider assessment and feedback, patient reminders) to integrate colorectal, breast, and cervical cancer screenings
Implementing	The Rhode Island Department of Health worked with 8 federally qualified health centers (one was WellOne Primary Medical and Dental Care). All colorectal cancer, breast, and cervical cancer screening funding to federally qualified health centers (FQHC) was provided under one contract, and patient navigation was implemented across the 8 FQHCs.	Pilot testing inclusion of breast and cervical cancer screening with patient navigation for colorectal cancer screening in one health center (WellOne Primary Medical and Dental Care)
Sustaining	Eligible men and women in Nebraska are screened for colorectal cancer through the state-funded Nebraska Colon Cancer Screening Program. Eligible women also receive breast, cervical, heart disease, and diabetes screening via Nebraska’s Every Woman Matters Program. Nebraska changed its payment model in 2018 from fee-for-service to fixed cost subawards.	Designing an optimal reimbursement approach for integrated payments for colorectal, breast, and cervical cancer screening to local health departments that administered the screening (19 in FY2018, 14 in FY2019, and 13 in FY2020)

Note: ^a^ID partnered with 6 health systems (5 health systems completed the checklist as a system, whereas 1 health system was an insurer with 2 demonstration clinics). Because each of these clinics was independently owned, they completed the checklist separately. We report on 7 sites

Fiscal Year (FY); FY2018 = 7/1/2017–6/30/2018; FY2019 = 7/1/2018–6/30/2019; FY2020 = 7/1/2019–6/30/2020

### Phases of implementation

The three programs focused on integration at different phases of implementation. ID-DHW, in their planning phase for integrated implementation strategies, conducted a systematic baseline assessment among their partner health systems to identify optimal approaches to promote cancer screenings. RI-DOH pilot-tested integrating breast and cervical cancer screening into their ongoing colorectal cancer screening navigation program during the implementation phase. NE-DHHS experimented to identify efficient integrated payment models, from fee-for-service to pay-for-performance approaches, for their partners who administered cancer screening in the sustainment phase.

### Program descriptions

#### Idaho Department of Health & Welfare

ID-DHW wanted to assess opportunities to integrate policies and evidence-based interventions to promote screening for colorectal, breast, and cervical cancers. Program staff were interested in the integration of cancer screening promotion activities to streamline logistics and improve efficiency for their partner health systems. ID-DHW also wanted to avoid burdening health systems with requests from multiple state programs doing similar work.

ID-DHW partnered with six health system partners. All health systems chose to implement evidence-based interventions for colorectal and breast cancer screenings, and five of the six also chose to implement evidence-based interventions for cervical cancer screening. To identify optimal approaches to integrate cancer screening promotion strategies, the ID-DHW developed a checklist in year 3 of the CRCCP and tailored it to integrated implementation in year 5 of the CRCCP (year 3 of the NBCCEDP) to assess the current state of policies and interventions to promote colorectal, breast, and cervical cancer screenings before their work with each health system.

#### WellOne Primary Medical and Dental Care, Rhode Island

RI-DOH partnered with eight FQHCs across the state via the RI-DOH Colorectal Cancer Prevention and Women’s Cancer Screening Programs. The aim of RI-DOH Colorectal Cancer Prevention is to increase colorectal cancer screening rates among persons aged 50 to 75 years within partner health systems [4]. The mission of RI-DOH Women’s Cancer Screening Programs is to reduce the number of cases and deaths from breast and cervical cancer, among women with limited access to health care [5]. One FQHC, WellOne Primary Medical and Dental Care, has four health centers in Rhode Island. We worked with one of its health centers to learn more about its patient navigation program. A key goal of the patient navigation program is to help patients overcome barriers to colorectal cancer screening [4, 6, 7] (from enrollment into the program to follow-up colonoscopy for a positive fecal immunochemical test [FIT]). WellOne Primary Medical and Dental Care piloted integrating breast and cervical cancer screening promotion into its colorectal cancer navigation program. The patient navigator would attempt to talk about several cancer screenings with a patient, when appropriate, during the same discussion.

#### Nebraska Department of Health and Human Services

NE-DHHS provides colorectal cancer screening services primarily through the Nebraska Colon Cancer Screening Program. During this study, Nebraska funded this program, not the CDC’s CRCCP. Eligible women also receive breast cancer and cervical cancer screenings through the Every Woman Matters program [8] using funding from CDC’s NBCCEDP. Every Woman Matters also provides services for heart disease and diabetes with funding from the Well-Integrated Screening and Evaluation for Women Across the Nation (WISEWOMAN); however, we do not evaluate these activities here. For cancer screenings, Every Woman Matters pays for office visits associated with Pap tests, pelvic exams, clinical breast exams, age-appropriate mammograms, and a limited number of diagnostic tests. For the Nebraska Colon Cancer Screening Program, NE-DHHS contracts with local health departments to distribute fecal occult blood tests (FOBT) to persons who are eligible (e.g., men and women who are uninsured, aged 50–74 years, and residents of Nebraska) and to follow up with residents who have not returned FOBTs or have had a positive FOBT. Local health departments are also responsible for identifying and navigating patients for breast and cervical cancer screenings.

Until 2018, NE-DHHS contracted with local health departments that paid for activities performed by the local health department related to colorectal, breast, and cervical cancer screenings. However, in 2018, NE-DHHS changed their contract structure to fixed cost subawards with pay-for-performance payments, meaning that NE-DHHS began to pay its local health departments for deliverables and outcomes (e.g., number of people recruited and number of screenings completed). We report screening process metrics, outcomes, and the costs of NE-DHHS administering the integrated payment model across colorectal, breast, and cervical cancer screening.

### Data collection and analysis

#### Idaho Department of Health & Welfare

We analyzed the ID-DHW Baseline Assessment Checklist (checklist), which ID-DHW developed and is detailed in Table 2. The complete checklist is found in Supplemental Appendix 1. The checklist functioned as a readiness assessment tool for the health systems. The tool included five categories for colorectal cancer screening, breast cancer screening, and cervical cancer screening: policies and processes, provider assessment and feedback, patient reminders, provider reminders, and reducing structural barriers. Each category had a specific number of components, which were activities completed by health systems. Once the health system completed the checklist, it could determine its capacity to implement the interventions included. We report on the average and the range of the percentage of components completed for promoting colorectal, breast, and cervical cancer screenings across health systems.

**Table 2:** Idaho department of health and welfare: description of baseline assessment checklist to promote colorectal, breast, and cervical cancer screening

Categories	Examples of components included
Policies and Processes	Screening rate is known at each site; colorectal, breast, and cervical cancer screening policies follow national guidelines
Provider Assessment & Feedback	Whether provider assessment and feedback rates are derived via electronic health records; whether reports are discussed with providers
Patient Reminders	Whether patient reminders are done through electronic health records; whether reminders are used for cancer screenings
Provider Reminders	How often providers are reminded to discuss screening with their patients; how they are reminded
Reducing Structural Barriers	Whether specific patient barriers (e.g., access to transportation, service delivery hours, knowledge and understanding of steps of screening tests) are addressed and documented

As mentioned, ID-DHW partnered with six health systems. Five of the health systems completed one checklist each. One health system was an insurer with two demonstration clinics; because each of these clinics was independently owned, the health system completed the checklist separately for each. Therefore, we report on analyzed checklist data from seven sites, five health systems and two clinics from one health system.

#### WellOne Primary Medical and Dental Care, Rhode Island

During the 2018 implementation period, we worked with a nurse-patient navigator at a federally qualified health center to collect details on patient navigation activities, number of patients navigated, information on patient barriers to screening (identified by patient navigator), as well as patient navigator time and cost. Patient navigation activities included identifying patients who were due for colorectal cancer screening; assessing whether these patients were also due for breast or cervical cancer screenings; reviewing patient records for previous referrals, documentation of screening conversation with provider, and risk status; contacting patients to discuss cancer screening and screening options; documenting contact in the electronic medical record; ordering or referring patients to screening; and following up with patients with no documentation on screening or with positive screening tests. By using salaries and times associated with various patient navigation activities, we calculated patient navigation cost per activity. We also collected patient navigation costs related to nonlabor activities, such as printing reminders and postage for mailings.

#### Nebraska Department of Health and Human Services

We collected information from the NE-DHHS on details about the annual revisions to the payment model to examine whether there were corresponding changes in screening outcomes and cost. We collected data for the following periods: Fiscal Year (FY) 2017 = 7/1/2016–6/30/2017; FY2018 = 7/1/2017–6/30/2018; FY2019 = 7/1/2018–6/30/2019; FY2020 = 7/1/2019–6/30/2020. For colorectal cancer screening, we collected data on the number of FOBT kits distributed and returned by year through follow-up across the health departments. For breast and cervical cancer screening, we collected the number of women who were identified to need screening by the health departments, how many were navigated, and how many were screened across the health departments. We derived program cost data, such as the costs of navigating and supporting patients through the screening process, and we calculated the screening promotion cost-per-person screened for breast and cervical cancer and separately for colorectal cancer. We did not include clinical cost of the screening tests or diagnostic procedures. In addition, we calculated cost-per-person screened at the awardee level for administrative, evaluation, quality improvement, and strategic planning activities (*administrative costs*). Lastly, we reported the total screening promotion cost per person screened for breast, cervical, and colorectal cancer combined. We presented colorectal cancer separately, but breast and cervical together, as this was how the program collected and reported data for NBCCEDP. The total cost per person screened was calculated by dividing the total cost paid to local health departments (not including costs for screening and diagnostic follow-up) divided by the number of persons screened.

#### Qualitative interviews

Two staff, one interviewer and one notetaker from Research Triangle Institute (RTI), conducted three structured interviews with people involved with the program in each of the three states. RTI conducted one interview per state with three interviewees from Idaho, one interviewee from Rhode Island, and one interviewee from Nebraska. Key informants included program directors, program managers, health center staff, and a patient navigator. The interviews were conducted remotely via Zoom during the spring and summer of 2020, and each lasted approximately 30 min. We asked informants about the integrated strategies for their cancer screening programs and about facilitators and barriers to the integration. The qualitative data were used in this paper to describe the programs and provide context to the quantitative data.

## Results

### Idaho Department of Health & Welfare

In Table 3, we show the percentage of total policy and intervention components in place before formal engagement in the seven sites in Idaho, and we report here in the results the overall averages of the analyzed checklist data. On average, the percentage of policy and process components in place by site for colorectal cancer was higher than for the other cancers. On average, the percentage of components addressed were 68.6% for colorectal cancer screening compared with 49.0% for breast cancer screening and 48.9% for cervical cancer screening. Similarly, for provider assessment and feedback, on average, 66.7% of the components were reported for colorectal cancer screening, 34.9% for breast cancer screening, and 51.8% for cervical cancer screening. Across sites, the percentages were similar for patient reminders with 57.9% addressed for colorectal cancer screening, 46.6% for breast cancer screening, and 54.4% for cervical cancer screening. Sites reported a similar percentage for provider reminders for each cancer screening: on average, sites addressed 54.0% of the components for colorectal cancer, 50.8% for breast, and 50.0% for cervical. Less than half of components for reduction of structural barriers were addressed across all three cancer screenings: on average, 40.8% were completed for colorectal cancer, 37.7% for breast, and 47.0% for cervical. Each individual site varied depending on their experience with implementing policies, processes, and evidence-based interventions to promote screening for multiple types of cancers.

**Table 3:** ID-DHW: percentage of total policies and interventions implemented at baseline by site (CRCCP program year 5, NBCCEDP program year 3)

	Cancer screening	Total components	Site 1	Site 2	Site 3	Site 4	Site 5	Site 6	Site 7	Overall average (range)
		(*N*)	(%)	(%)	(%)	(%)	(%)	(%)	(%)	
Policies and Processes	Colorectal	30	63.3 (19)	83.3 (25)	80.0 (24)	73.3 (22)	90.0 (27)	80.0 (24)	10.0 (3)	68.6 (10.0–90.0)
	Breast	28	53.6 (15)	67.9 (19)	67.9 (19)	60.7 (17)	14.3 (4)	71.4 (20)	7.1 (2)	49.0 (7.1–71.4)
	Cervical	29	n/a	72.4 (21)	72.4 (21)	55.2 (16)	13.8 (4)	72.4 (21)	6.9 (2)	48.9 (6.9–72.4)
Provider Assessment & Feedback	Colorectal	9	44.4 (4)	88.9 (8)	100.0 (9)	88.9 (8)	88.9 (8)	22.2 (2)	33.3 (3)	66.7 (22.2–100.0)
	Breast	9	44.4 (4)	33.3 (3)	44.4 (4)	33.3 (3)	33.3 (3)	22.2 (2)	33.3 (3)	34.9 (22.2–44.4)
	Cervical	9	n/a	33.3 (3)	100.0 (9)	88.9 (8)	33.3 (3)	22.2 (2)	33.3 (3)	51.8 (22.2–100.0)
Patient Reminders	Colorectal	19	10.5 (2)	73.7 (14)	78.9 (15)	68.4 (13)	63.2 (12)	100 (19)	10.5 (2)	57.9 (10.5–100.0)
	Breast	19	10.5 (2)	73.7 (14)	57.9 (11)	57.9 (11)	36.8 (7)	78.9 (15)	10.5 (2)	46.6 (10.5–78.9)
	Cervical	19	n/a	73.7 (14)	68.4 (13)	63.2 (12)	36.8 (7)	73.7 (14)	10.5 (2)	54.4 (10.5–73.7)
Provider Reminders	Colorectal	9	77.8 (7)	77.8 (7)	44.4 (4)	77.8 (7)	55.6 (5)	44.4 (4)	0	54.0 (0–77.8)
	Breast	9	77.8 (7)	77.8 (7)	22.2 (2)	77.8 (7)	55.6 (5)	44.4 (4)	0	50.8 (0–77.8)
	Cervical	9	n/a	77.8 (7)	44.4 (4)	77.8 (7)	55.6 (5)	44.4 (4)	0	50.0 (44.4–77.8)
Reducing Structural Barriers	Colorectal	14	7.1 (1)	71.4 (10)	78.6 (11)	57.1 (8)	21.4 (3)	50 (7)	0	40.8 (7.1–78.6)
	Breast	11	0	81.8 (9)	81.8 (9)	63.6 (7)	0	36.4 (4)	0	37.7 (0–81.8)
	Cervical	11	n/a	81.8 (9)	81.8 (9)	81.8 (9)	0	36.4 (4)	0	47.0 (0–81.8)

Note: ID partnered with six health systems. Five health systems completed the checklist as a system, whereas one health system was an insurer with two demonstration clinics. Because each of these clinics was independently owned, they completed the checklist separately. We report on seven sites. Numerators are to the right of percentages in parentheses

According to ID-DHW stakeholders, there were several program elements that facilitated the initiation of cancer screening prevention integration after their planning phase. The first of these elements was a pre-implementation checklist for health clinics to determine their capacity for implementing each type of cancer screening and undertake specific enhancements for coordinated and integrated screenings to be initiated. The second of these program elements was having administrative support and buy-in from the chief executive officers and operating officers, providers, and support staff. Barriers to integration focused mainly on staff: staff capacity to implement interventions and staff turnover. Lack of adequate resources related to staff and electronic medical record enhancements in clinics was also cited as impeding implementation. In Supplemental Appendix 2, we provide an overview of facilitators and barriers to integrating policies and interventions by program.

### WellOne Primary Medical and Dental Care, Rhode Island

We described patient navigation services provided and patient barriers to screening in Table 4. The predominant navigation service was for colorectal cancer screening: of all services, more than three-quarters (76.1%, 779/1023) were only for colorectal cancer screening. Nearly 17% (172/1023) of navigation services were for multiple cancer screenings, such as colorectal, breast, or cervical cancers. Patient navigation services for only breast cancer accounted for 4.8% (49/1023) of services provided, follow-up for diagnostic testing for positive screening results accounted for 1.5% (15/1023) of services, and navigation for only cervical cancer screening accounted for less than 1% of services provided.

**Table 4:** WellOne primary medical and dental care, Rhode Island: description of patient navigation services and patient barriers to cancer screening

	Implementation (2018) (%)
Patient navigation cancer screening services provided (*N*=1023)	
Colorectal cancer screening only	76.1(779)
Multiple cancer screenings (e.g., colorectal cancer, cervical, and breast cancer screenings)	16.8 (172)
Breast cancer screening only	4.8 (49)
Follow-up diagnostic testing for screen positives^a^	1.5 (15)
Cervical cancer screening only	0.8 (8)
Patient barriers to cancer screening identified by patient navigator (*N*=148)	
Financial barriers or insurance coverage issues	30.4 (45)
Psychosocial issues (including fear)	23.6 (35)
Transportation issues	23.6 (35)
Family/community support issues	6.8 (10)
Medical and mental health comorbidity or disability	6.8 (10)
Work schedule conflicts	6.8 (10)
System problems with scheduling care	1.4 (2)
Literacy or language barriers	0.7 (1)
Total cost of implementation in 2018 (labor and nonlabor)^b^	$28,160
Identify patients due for screening via list or provider referral	1077
Review patient record for previous referrals, documentation of screening conversation with provider, high-risk status	2992
Contact patient to discuss colorectal cancer and other cancer screenings when appropriate	10,293
By phone	7899
By mail	2394
Document contact in Electronic Health Records	3112
Ordering/referring patients to screenings	1317
Following up with patients if no screening documented	1915
Following up with patients if positive screen	957
Nonlabor costs (e.g., materials, supplies, printing)	6497

Notes: ^a^Few positive FIT results were reported. ^b^Total cost refers to program costs and excludes cost of screening and diagnostic follow-up

However, the perspective of the patient navigator we spoke with was different. The patient navigator indicated it was simpler to discuss numerous screening tests and preventive measures during one call, as it was easier for the patient navigator to go through and discuss any and all testing a patient may need. Nonetheless, the patient navigator indicated that it was sometimes challenging for a patient to hear the list. It could be overwhelming if a patient was not prepared or did not initially understand the purpose of the tests or screenings. The patient navigator showed it was always better to have a conversation with a patient once a provider had discussed the tests or screenings first.

The patient navigator identified barriers (*N*=148) to patients being screened for colorectal cancer or other cancers. The largest categories of barriers identified included financial or insurance issues (30.4%, 45/148); psychosocial issues, such as fear of the test and fear of test outcome (23.6%; 35/148); and transportation (23.6%; 35/148).

We also reported the cost of patient navigation activities in Table 4. The total cost of implementing a patient navigation program in 2018 was $28,160. The largest portion of the cost ($10,293) was contacting patients to discuss cancer screenings. More time and costs were related to contacting patients by phone ($7899) than by mail ($2394). About $3000 was allocated to documenting the contacts in the electronic medical records ($3112) and reviewing the patient record before contact ($2992). The total amount attributed to nonlabor costs was $6947, which included materials, supplies, and printing.

In total, 105 screening colonoscopy appointments were made and 48 tests taken, a completion rate of 45.7% (data not shown). In addition, 163 FIT and Cologuard kits were distributed and 74 were returned, a 45.4% completion rate (data not shown). In total, navigation to complete diagnostic colonoscopy was also provided for six patients (data not shown). Breast and cervical navigation constituted a very small proportion of the patient navigation activities, and completion rates were not tracked.

### Nebraska Department of Health & Human Services

The descriptions of the integrated payment model, screening outcomes, and cost by year are shown in Table 5. In fiscal year (FY) 2017, the payment model was fee-for-service and included a 25% administrative fee and a payment-per-service for 19 local health departments. During FY2018 through FY2020, the payment model was changed to a fixed cost subaward. The administrative cost was eliminated, and a navigation payment capped at $208 per person was put in place. In addition, payment for meeting screening targets was added to encourage screening completion under a pay-for-performance structure. Although 19 local health departments participated in FY2018, this number decreased to 14 in FY2019 and 13 in FY2020.

**Table 5:** NE-DHHS: description of integrated payment model, screening outcomes, and cost by year

	FY2017 (7/1/2016–6/30/2017)	FY2018 (7/1/2017–6/30/2018)	FY2019 (7/1/2018–6/30/2019)	FY2020 (7/1/2019–6/302020)
Description of integrated payment model				
Contract	Fee-for-dervice	Fixed cost subaward	Fixed cost subaward	Fixed cost subaward
FIT kit distribution and navigation payment for colorectal cancer screenings	Payment per service; % paid for kit return	Fixed fees for FIT kit distribution and kit returned		
Identification and navigation payment for breast and cervical cancer screening	Payment per service	Capped at $208 per person navigated		
Payment for meeting targets (separate payments for colorectal and breast/cervical cancer screenings)	None	Pay for performance^a^		
Administrative cost (across all cancer screenings)	25% of total	Eliminated		
Strategic planning and quality improvement fee (across all cancer screenings)			$2000 per quarter	
Integration across programs	Administrative cost	Pay-for-performance approach		
Local health department (LHD) implementing partners				
Number of local health departments participating in the program	19	19	14	13
Screening outcomes				
Colorectal cancer				
FOBT kit distribution	4101	3418	2308	1201
Kits returned (individuals screened)	2162	1867	1037	703
Return rates (%)	52.7	54.6	44.9	58.5
Breast and cervical cancers				
Flagged	1590	866	356	374
Navigated	301	292	283	291
Screened	198	265	262	277
% screened screened/reach	12.5%	30.6%	73.6%	74.1%
% screened screened/navigated	65.8%	90.8%	92.6%	95.2%
Cost^b^				
Cost per person screened for colorectal cancer (includes FIT kit distribution, navigation and payment for meeting screening targets)	23	33	44	42
Cost per person screened for breast and cervical cancer (includes patient identification, navigation and payment for meeting screening targets)	1173	452	603	508
Cost per person screened for administrative, evaluation, quality improvement and strategic planning	112	–	19	38
Total promotion costs per person screened for breast, cervical and colorectal cancer	389	85	195	234

^a^Up to 30% for completion rate for breast and cervical navigation and up to 20% for completion rate for colorectal cancer screening

^b^Cost of FIT kit tests, screening tests, and diagnostic procedures are not included. Therefore, the costs presented in the table do not include clinical cost of delivering screening

*FY*, fiscal year

In FY2017, the FOBT return rate was 52.7% (2162/4101). Beginning in FY2018, there was no clear trend for colorectal cancer uptake across health departments: it increased to 54.6% (1867/3418) in FY2018, decreased to 44.9% (1037/2308) in FY2019, and increased again to 58.5% (703/1201) in FY2020.

In FY2017, there were 1590 women who were eligible for breast or cervical cancer screening and 12.5% (198/1590) were screened. Of the women who were navigated, 65.8% (198/301) were screened. Under the fixed cost subaward, the trend for breast and cervical screening improved. There was an increase in uptake for those screened who were reached (ranging from 30.6% [265/866] in FY2018 to 74.1% [277/374] in FY2020) and for those screened who were navigated (ranging from 90.8% [265/292] in FY2018 to 95.2% [277/291] in FY2020).

We also presented costs per person screened in Table 5. Cost per person screened (includes FIT kit distribution, navigation, and payment for meeting screening targets) for colorectal cancer was $23 in FY2017 under FFS and increased from $33 in FY2018 to $44 in FY2019 under the fixed cost subaward. Cost per person screened for colorectal cancer decreased slightly in FY2020 to $42. Under fee-for-service in FY2017, the cost (for identification, navigation, and payment for meeting screening targets) per person screened for breast and cervical cancer was $1173. The costs decreased under the fixed cost subaward to $452, $603, and $508 in FY2018, FY2019, and FY2020, respectively. Administrative cost per person screened was highest in FY2017 under fee-for-service at $112 per person screened. There were no administrative costs in FY2018 under the fixed cost subaward, and the administrative cost per person was $19 in FY2019 and $38 in FY2020. Total promotion costs per person screened for breast, cervical, and colorectal cancer were highest in FY2017 at $389 under FFS and ranged from $85 in FY2018 to $234 in FY2020.

In Nebraska, there were benefits to integrating cancer screening contracts. According to one NE-DHHS stakeholder, integrating contracts allowed for a more comprehensive set of services to be delivered at one time to individuals. Integration of cancer screening services streamlined the processes for risk assessment and patient navigation services that were appropriate for each individual’s unique needs (e.g., cultural, health literacy, health risk). Integration also decreased the total resources needed to deliver services at both the state and local levels. For example, in integrating breast, cervical, and colorectal cancer screenings, there would be only one contract to manage instead of three (CRCCP, NBCCEDP, and WISEWOMAN), and only one person would be needed to provide technical assistance to subawardees instead of three separate program staff. There were downsides to cancer screening integration, as well. It was possible that in some local health departments, staff might have had an interest in promoting one service over another, instead of promoting all services equally. This may have translated into more cancer screening tests for one type of cancer compared to others, instead of equal numbers of screenings for all cancers (with consideration to age and gender). In addition, administratively, it was difficult when budget periods were different, as funding streams had different contract start and end dates.

## Discussion

Our study evaluated approaches to integrate implementation of cancer screening interventions at different phases of program delivery to assess optimal approaches for planning, implementing, and sustaining interventions to increase screening uptake. Integration of cancer screenings, such as FIT and flu shots as well as mammograms and FIT, has shown some success in improving CRC screening uptake [9–11]. Our findings indicate that there are definite benefits to be gained by coordinating across cancer screening and integrating processes, but each phase, whether planning and initiation, ongoing implementation, or sustaining integrated screening, all had challenges, as well. Therefore, our conclusion from the case study assessments presented in this study is that integrated cancer screening interventions can be efficient, but additional research is needed to assess whether the benefits clearly outweigh potential drawbacks.

Our findings offer new data to support the recommendations from the 2012 Institute of Medicine’s report, *Primary Care and Public Health: Exploring Integration to Improve Population Health*, which also identified both barriers and facilitators related to integration [12]. Integration can lead to patient-centered care, but this is also a complex health care issue, and prior studies, similar to our recommendation, have also called for systematic empirical studies to test the values of alternative approaches [13, 14]. We recommend that additional evaluations be conducted by using implementation science frameworks to assess context, moderating factors, and outcomes to quantify a comprehensive set of benefits and drawbacks to integrated delivery of screening strategies and other interventions. Integrated delivery is being attempted in various contexts, including pediatric behavior health services [15–17], chronic diseases, and rural settings. Lessons learned from these initiatives and studies will offer shared guidance for moving the field forward to achieve the promise of efficiency, while ensuring improved patient experience and outcomes.

In Idaho, there were similar components in policies and processes and evidence-based interventions (patient reminder, provider reminder, and addressing structural barriers) for promotion of colorectal, breast, and cervical cancer screening. As a whole, similar components were addressed at the sites for patient reminder, provider reminder, and addressing structural barriers, which shows that integration is feasible for these interventions across the multiple cancers. The components for provider assessment and feedback addressed varied across colorectal, breast, and cervical cancer screening, with the largest component addressed for colorectal cancer. One possible reason for the focus on colorectal cancer screening is the funding and technical support provided through the CRCCP [2]. Although health systems are likely able to assess and streamline policies across multiple cancer screenings, challenges related to capacity, resources, and staff turnover remain barriers to successful implementation of integrated evidence-based interventions.

At WellOne Primary Medical and Dental Care in Rhode Island, the integration of navigation for breast and cervical cancer screening into an existing colorectal cancer screening program yielded mixed results. Fewer patient navigation services were provided for breast and cervical cancer screening than for colorectal cancer screening. On the basis of study findings, we believe there are several possible reasons for this, including the underlying prioritization of colorectal cancer screenings with navigation procedures implemented for a longer time, as the patient navigator was funded by CRCCP; not all individuals were eligible (or due) for multiple cancer screenings; and the patient not wanting to discuss all cancer screenings they were due for or eligible to receive. In addition, there were differences in the NBCCEDP and CRCCP funding cycle starting periods, and patient navigation data collection requirements differed. There is likely a need for a more focused approach to have a truly integrated navigation program for colorectal, breast, and cervical cancer screening. Although there were clear efficiencies identified from the health center and navigator perspectives, it is not clear whether the patients always viewed the discussion about multiple cancer screenings as a benefit. We recommend that comprehensive assessment of patient wishes and preferences for integrated cancer and other screenings be conducted to design programs that will offer benefits to all stakeholders involved.

Data from Nebraska suggest that although NE-DHHS attempted to integrate cancer screenings (colorectal with breast and cervical) through its pay-for-performance funding, there are some aspects that are unique to the type of disease that still require individual payment structures. For example, FIT kit distribution for colorectal cancer is very different from referral for mammograms and provision of human papillomavirus deoxyribonucleic acid (HPV DNA) or Pap smear screening for cervical cancer. Integration did allow NE-DHHS to provide one payment for administrative cost and a similar approach for pay-for-performance model. However, the lesson learned from the fixed cost payment model with pay-for-performance is that the screening completion rates generally improved and cost per person successfully screened was lower, but reach of the program was overall lower as fewer local health departments participated because of the perceived reduction in funding support. There was also variation across cancer screening with the number of colorectal cancer screenings declining and the number of breast and cervical screening increasing. Some of this variation could be because FIT kits were distributed only during spring each year, whereas the other screenings occurred throughout the year and were less likely to be affected by weather and severe flooding that previously occurred in the springtime. NE-DHHS initiated payment reform to identify an optimal model, and lessons learned will be applied to further tailor the reimbursement model to optimize both reach and screening completion.

Our findings are subject to a few limitations. First, the three programs may not be representative of the 30 CRCCP programs and other health systems implementing cancer screening; thus, the results are not generalizable. In addition, these case studies were conducted to offer a snapshot of the costs, benefits, and challenges of integrated delivery of cancer screening, and we did not perform systematic evaluations at the clinic or program level. Lastly, our study only focused on multiple cancer screenings and did not include other chronic conditions, such as diabetes assessment, hypertension screening, and weight management, which are often also integrated.

## Conclusions

For this research, we report on three case studies on the integrated implementation of cancer screening interventions, the results of which offer insight into the potential role of this approach in offering patient-centered care that can improve health outcomes and also increase efficiency. The case studies provide models that can be further evaluated to identify optimal approaches that should be adopted to improve cancer screening uptake and to efficiently use available resources. Health systems also will need additional resources to help in the planning and evaluation of interventions using implementation science methods to improve integration of cancer screenings. Lessons learned from integrated implementation of cancer screening interventions could be relevant to other preventive measures, including diabetes testing, hypertension screening, tobacco cessation, and weight management. Our future data collection efforts will systematically evaluate integrated approaches, including the costs of these approaches.

## Supplementary Information

**Additional file 1: Supplemental Appendix 1.** Pre-Implementation: Evidence-based Intervention Checklist. This document is Idaho’s complete checklist.

**Additional file 2: Supplemental Appendix 2**. Facilitators and barriers to integrating policies, interventions, and strategies across cancer screening programs identified from informant interviews.

## Data Availability

The underlying data generated during the study are not publicly available as health system participants cannot be adequately masked and only summarized data was available to the evaluation team. Data were collected by RTI International under contract with the Centers for Disease Control and Prevention.

## Abbreviations

CDC: Centers for Disease Control and Prevention
CRCCP: Colorectal Cancer Control Program
FOBT: Fecal occult blood tests
FQHC: Federally qualified health center
FIT: Fecal immunochemical test
FY: Fiscal year
ID-DHW: Idaho Department of Health and Welfare
NBCCEDP: National Breast and Cervical Cancer Early Detection Program
NE-DHHS: Nebraska Department of Health and Human Services
RI-DOH: Rhode Island Department of Health
WISEWOMAN: Well Integrated Screening and Evaluation for Women Across the Nation
