# Supplementary material for: Improving the efficiency of integrated cancer screening delivery across multiple cancers: case studies from Idaho, Rhode Island, and Nebraska
Source: Implement Sci Commun. 2022 Dec 16;3:133. doi: 10.1186/s43058-022-00381-4 (PMC9756516; doi:10.1186/s43058-022-00381-4)
Supplement: Supplementary file 1 — Additional file 1: Supplemental Appendix 1. Pre-Implementation: Evidence-based Intervention Checklist. This document is Idaho’s complete checklist. [file 43058_2022_381_MOESM1_ESM.docx]

**Pre-Implementation: Evidence-based Intervention Checklist**

Table of Contents

[Health System Name 2](#_Toc19606879)

[Project Team 2](#_Toc19606880)

[Policies and Processes 3](#_Toc19606881)

[Provider Assessment & Feedback 6](#_Toc19606882)

[Patient Reminders 8](#_Toc19606883)

[Provider Reminders 10](#_Toc19606884)

[Reducing Structural Barriers 11](#_Toc19606885)

**Instructions**The purpose of this checklist is to identify progress already achieved to increase breast, cervical, colorectal screening and/or HPV vaccination rates within your health system. This information will be used to identify next steps, including strategies and activities to inform your workplan. The checklist is incremental in that within each section the basic steps are listed first with more advanced implementation listed thereafter. The minimum elements of each intervention are provided, those elements that are shaded in blue are considered supplemental. For elements that have multiple options (ex. Reminder cards are sent via: email, letter, text or phone call) at least one of the options must be included/addressed to ensure full intervention implementation. The checklist will also be completed at the end of the funding period to measure progress from baseline.

Please complete the EBI Checklist with input from the full cancer health system project team. Choose a response from the drop-down box provided in each cell and provide any comments that explain current practices further in the Notes column.

**Submit one copy per Health System by November 1, 2019**

# Health System Name

Click or tap here to enter text.

# Project Team

Each individual identified in your grant application should be listed below.

| **Role** | **Key Person Name** | **Job title** |
| --- | --- | --- |
| **Cancer Screening Champion**  (may overlap with another role below) |  |  |
| **HPV Vaccination Champion**  (if applicable, may overlap with another role below) |  |  |
| **Quality Improvement** |  |  |
| **Health Information Technology** |  |  |
| **Clinical Representative**  (e.g. Nurse, Medical Assistant or Care Coordinator) |  |  |
| **Organizational Decision Maker**  (e.g. COO or CEO) |  |  |
| **Grant Coordinator** |  |  |
| **Other key staff** *optional*  (e.g. patient navigators) |  |  |

# Policies and Processes

Choose a response from the drop-down box provided in each cell and provide any comments that explain current practices further in the Notes column.

| **QI PRIORITIES & DATA** | **Breast**  **Cancer**  **Screening** | **Cervical**  **Cancer Screening** | **Colorectal Cancer Screening** | **HPV Vaccination** | **Notes** |
| --- | --- | --- | --- | --- | --- |
| Is a quality improvement priority for your health system (note metric definition used and how it is identified as a priority in the Notes column). | Choose an item. | Choose an item. | Choose an item. | Choose an item. |  |
| The screening rate of the health system and each clinic site is known. | Choose an item. | Choose an item. | Choose an item. | Choose an item. |  |

| **PATIENT RISK ASSESSMENT** | **Breast**  **Cancer**  **Screening** | **Cervical**  **Cancer Screening** | **Colorectal Cancer Screening** | **HPV Vaccination** | **Notes** |
| --- | --- | --- | --- | --- | --- |
| There is a standard process for assessing a patient’s risk for: | Choose an item. | Choose an item. | Choose an item. |  |  |
| The process includes assessment and documentation of: |  |  |  |  |  |
| Age | Choose an item. | Choose an item. | Choose an item. |  |  |
| Family history | Choose an item. | Choose an item. | Choose an item. |  |  |
| Other Health Conditions | Choose an item. | Choose an item. | Choose an item. |  |  |
| All relevant clinic staff receive training on the patient risk assessment process. | Choose an item. | Choose an item. | Choose an item. |  |  |

| **POLICY** | **Breast**  **Cancer**  **Screening** | **Cervical**  **Cancer Screening** | **Colorectal Cancer Screening** | **HPV Vaccination** | **Notes** |
| --- | --- | --- | --- | --- | --- |
| There is a standard process for screening/HPV vaccination. | Choose an item. | Choose an item. | Choose an item. | Choose an item. |  |
| The policy follows national guidelines: (Note the national guidelines that are followed in the notes, and if there are deviations). | Choose an item. | Choose an item. | Choose an item. | Choose an item. |  |
| The policy indicates who should be screened/vaccinated for HPV | Choose an item. | Choose an item. | Choose an item. | Choose an item. |  |
| The policy details screening options as applicable. |  | Choose an item. | Choose an item. |  |  |
| The policy details screening frequency. | Choose an item. | Choose an item. | Choose an item. |  |  |
| A high sensitivity guaiac-based Fecal Occult Blood Test (FOBT) or Fecal Immunochemical Test (FIT) kit is available as a screening option (Note which tests are used within your clinics). |  |  | Choose an item. |  |  |
| All relevant clinic staff receive training on the policy (Note how the policy is distributed and type of training that occurs.). | Choose an item. | Choose an item. | Choose an item. | Choose an item. |  |
| The policy is signed by the CEO or Medical Director. | Choose an item. | Choose an item. | Choose an item. | Choose an item. |  |
| The policy is reviewed at least annually. | Choose an item. | Choose an item. | Choose an item. | Choose an item. |  |

| **WORKFLOW** | **Breast**  **Cancer**  **Screening** | | **Cervical**  **Cancer Screening** | **Colorectal Cancer Screening** | | **HPV Vaccination** | **Notes** |
| --- | --- | --- | --- | --- | --- | --- | --- |
| There is a standard procedure for cancer screening/HPV vaccination. | Choose an item. | Choose an item. | | Choose an item. | Choose an item. | |  |
| The procedure includes: (Note your procedure steps or attach a copy of your current procedure. Also note key staff involved in each step)   - Recommendation for cancer screening/HPV vaccination is made to appropriate patients |  | | | | | |  |
|  | Choose an item. | | Choose an item. | Choose an item. | | Choose an item. |  |
| - Cancer screening options are described to patient | Choose an item. | | Choose an item. | Choose an item. | |  |  |
| - Nature of test and any test preparation is described to the patient |  | | Choose an item. | Choose an item. | |  |  |
| - Referral process for procedures to be completed outside of the clinic | Choose an item. | | Choose an item. | Choose an item. | |  |  |
| - Documentation process for cancer screening/HPV vaccination recommendation (e.g. recommendation made but declined | Choose an item. | | Choose an item. | Choose an item. | | Choose an item. |  |
| - Documentation process for completed screening/vaccination administration (Note: for colorectal cancer includes type of screening completed, date and test results) | Choose an item. | | Choose an item. | Choose an item. | | Choose an item. |  |
| - Follow up process for incomplete cancer screening or referral | Choose an item. | | Choose an item. | Choose an item. | |  |  |
| - Follow up process to ensure HPV vaccination series completion |  | |  |  | | Choose an item. |  |
| - Follow up steps for both positive and negative test results. | Choose an item. | | Choose an item. | Choose an item. | |  |  |
| All relevant clinic staff receives training about the procedure (Note how the procedure was distributed and type of training that occurred). | Choose an item. | | Choose an item. | Choose an item. | | Choose an item. |  |
| Patients are counseled about the need for screening/HPV vaccination:   - During routine visits | Choose an item. | | Choose an item. | Choose an item. | | Choose an item. |  |
| - During sick or unexpected visits | Choose an item. | | Choose an item. | Choose an item. | | Choose an item. |  |
| - Outside of office visit | Choose an item. | | Choose an item. | Choose an item. | | Choose an item. |  |
| - Other (Describe in notes section) | Choose an item. | | Choose an item. | Choose an item. | | Choose an item. |  |

# Provider Assessment & Feedback

Provider assessment and feedback interventions evaluate provider performance in delivering or offering screening to clients (assessment) and present providers with information about their performance in providing screening services (feedback). Feedback may describe the performance of a group of providers (e.g., mean performance for a practice), or an individual provider, and may be compared with a goal or standard. A provider can be generalized to any clinical staff providing direct care to patients.

Choose a response from the drop-down box provided in each cell and provide any comments that explain current practices in the Notes column.

| **PROVIDER ASSESSMENT & FEEDBACK** | **Breast**  **Cancer**  **Screening** | **Cervical**  **Cancer Screening** | **Colorectal Cancer Screening** | **HPV Vaccination** | **Notes** |
| --- | --- | --- | --- | --- | --- |
| Provider/care team level data can be extracted from the EHR | Choose an item. | Choose an item. | Choose an item. | Choose an item. |  |
| Provider/clinic team level data are available on:   - Screening/HPV vaccination rates - Patients who need screening/HPV vaccination - Missed opportunities/other feedback measures at this level. (Note measures e.g. missed opportunity at point of care) |  |  |  |  |  |
|  | Choose an item. | Choose an item. | Choose an item. | Choose an item. |  |
|  | Choose an item. | Choose an item. | Choose an item. | Choose an item. |  |
|  | Choose an item. | Choose an item. | Choose an item. | Choose an item. |  |
| Provider level reports on screening/HPV vaccination rates are routinely pulled. (Note how often reports are pulled) | Choose an item. | Choose an item. | Choose an item. | Choose an item. |  |
| Provider level reports on screening/HPV vaccination are routinely shared with providers and clinic staff (Note how data is shared and how often). | Choose an item. | Choose an item. | Choose an item. | Choose an item. |  |
| Reports are discussed with providers and clinic staff. | Choose an item. | Choose an item. | Choose an item. | Choose an item. |  |
| Reports are used by providers and clinic staff to change their behavior related to screening/HPV vaccination. | Choose an item. | Choose an item. | Choose an item. | Choose an item. |  |
| Providers and staff have ongoing discussions about how the screening/HPV vaccination system is working. | Choose an item. | Choose an item. | Choose an item. | Choose an item. |  |

# Patient Reminders

Patient reminders are written (letter, postcard, e-mail) or telephone messages (including automated messages) advising people that they are due for screening. These interventions can be untailored to reach the overall target population or tailored with the intent to reach one specific person, based on characteristics unique to that person, related to the outcome of interest, and derived from an individual assessment.

Choose a response from the drop-down box provided in each cell and provide any comments that explain current practices in the Notes column.

| **PATIENT REMINDERS - INFRASTRUCTURE** | **Breast**  **Cancer**  **Screening** | **Cervical**  **Cancer Screening** | **Colorectal Cancer Screening** | **HPV Vaccination** | **Notes** |
| --- | --- | --- | --- | --- | --- |
| Electronic Health Record is used.  (Note type and for how long) | Choose an item. | Choose an item. | Choose an item. | Choose an item. |  |
| Electronic Health Record is able to identify patients’ due for screening/HPV vaccination. | Choose an item. | Choose an item. | Choose an item. | Choose an item. |  |
| There is a patient reminder system in place for preventive services. | Choose an item. | Choose an item. | Choose an item. | Choose an item. |  |
| The patient reminder system is used for screening/HPV vaccination. | Choose an item. | Choose an item. | Choose an item. | Choose an item. |  |
| The patient reminder system notifies patients when they are past due for screening/HPV vaccination. | Choose an item. | Choose an item. | Choose an item. | Choose an item. |  |
| The patient reminder system notifies patients prior to screening/HPV vaccination being due (Note timeline for when they are reminded). | Choose an item. | Choose an item. | Choose an item. | Choose an item. |  |
| The patient reminder system notifies patients when screening or vaccination discussed but incomplete (Note the number of follow-up reminders and timeline for these). | Choose an item. | Choose an item. | Choose an item. |  |  |

| The patient reminder system notifies patients by:   - - Mail | Choose an item. | Choose an item. | Choose an item. | Choose an item. |  |
| --- | --- | --- | --- | --- | --- |
| - - Telephone (verbal) (Note if recording or completed by staff) | Choose an item. | Choose an item. | Choose an item. | Choose an item. |  |
| - - Telephone (text) | Choose an item. | Choose an item. | Choose an item. | Choose an item. |  |
| - - Electronically (email or patient portal) | Choose an item. | Choose an item. | Choose an item. | Choose an item. |  |

| **PATIENT REMINDERS - MESSAGES** | **Breast**  **Cancer**  **Screening** | **Cervical**  **Cancer Screening** | **Colorectal Cancer Screening** | **HPV Vaccination** | **Notes** |
| --- | --- | --- | --- | --- | --- |
| There is standard messaging provided to patients who need to be screened/vaccinated for HPV. | Choose an item. | Choose an item. | Choose an item. | Choose an item. |  |
| Standard messaging addresses:   - - Age at which to be screened/vaccinated for HPV | Choose an item. | Choose an item. | Choose an item. | Choose an item. |  |
| - - Affordability | Choose an item. | Choose an item. | Choose an item. | Choose an item. |  |
| - - Screening options | Choose an item. | Choose an item. | Choose an item. |  |  |
| - - Screening can and should occur even without symptoms | Choose an item. | Choose an item. | Choose an item. |  |  |
| - - Screening/vaccination can prevent cancer | Choose an item. | Choose an item. | Choose an item. | Choose an item. |  |
| Materials are written at a 5^th^ grade reading level. | Choose an item. | Choose an item. | Choose an item. | Choose an item. |  |
| Materials are culturally and linguistically appropriate for the patient population. | Choose an item. | Choose an item. | Choose an item. | Choose an item. |  |

# Provider Reminders

Provider reminders inform health care providers it is time for a client’s cancer screening test or vaccination (called a “reminder”) or that a client is overdue for screening or vaccination (called a “recall”).

Choose a response from the drop-down box provided in each cell and provide any comments that explain current practices in the Notes column.

| **PROVIDER REMINDERS** | **Breast**  **Cancer**  **Screening** | **Cervical**  **Cancer Screening** | **Colorectal Cancer Screening** | **HPV Vaccination** | **Notes** |
| --- | --- | --- | --- | --- | --- |
| There is a provider reminder system in place. | Choose an item. | Choose an item. | Choose an item. | Choose an item. |  |
| Provider reminders occur:   - Weekly | Choose an item. | Choose an item. | Choose an item. | Choose an item. |  |
| - Daily | Choose an item. | Choose an item. | Choose an item. | Choose an item. |  |
| - Immediately at patient point of care | Choose an item. | Choose an item. | Choose an item. | Choose an item. |  |
| - Other (Describe in notes section.) | Choose an item. | Choose an item. | Choose an item. | Choose an item. |  |
| Providers are reminded via:   - Staff huddles | Choose an item. | Choose an item. | Choose an item. | Choose an item. |  |
| - EHR chart prompts | Choose an item. | Choose an item. | Choose an item. | Choose an item. |  |
| - Preventive care checklists or record sheets | Choose an item. | Choose an item. | Choose an item. | Choose an item. |  |
| - Other (Describe in notes section.) | Choose an item. | Choose an item. | Choose an item. | Choose an item. |  |

# Reducing Structural Barriers

Structural barriers are non-economic burdens or obstacles that make it difficult for people to access cancer screening or HPV vaccination (e.g., inconvenient clinic hours, access to screening services).

Choose a response from the drop-down box provided in each cell and provide any comments that explain current practices in the Notes column.

| **ASSESSING & ADDRESSING BARRIERS** | **Breast**  **Cancer**  **Screening** | **Cervical**  **Cancer Screening** | **Colorectal Cancer Screening** | **HPV Vaccination** | **Notes** |
| --- | --- | --- | --- | --- | --- |
| There is a standard way to assess patient barriers to screening/HPV vaccination. | Choose an item. | Choose an item. | Choose an item. | Choose an item. |  |
| Patient barriers to screening are assessed:   - Geographic location | Choose an item. | Choose an item. | Choose an item. | Choose an item. |  |
| - Access to transportation | Choose an item. | Choose an item. | Choose an item. | Choose an item. |  |
| - Knowledge/understanding of test steps/HPV vaccination | Choose an item. | Choose an item. | Choose an item. | Choose an item. |  |
| - Service delivery hours | Choose an item. | Choose an item. | Choose an item. | Choose an item. |  |
| - Other (Describe in notes section.) | Choose an item. | Choose an item. | Choose an item. | Choose an item. |  |
| Patient barriers are documented. | Choose an item. | Choose an item. | Choose an item. | Choose an item. |  |
| Patient barriers are considered in screening recommendation and care coordination. | Choose an item. | Choose an item. | Choose an item. | Choose an item. |  |
| Provide FOBT/FIT kits as a screening option:   - During patient visits | Choose an item. | Choose an item. | Choose an item. | Choose an item. |  |
| - Pick up at front desk | Choose an item. | Choose an item. | Choose an item. | Choose an item. |  |
| - Direct mail to patients | Choose an item. | Choose an item. | Choose an item. | Choose an item. |  |
| Patient barriers are addressed to meet patient needs (Note how this occurs). | Choose an item. | Choose an item. | Choose an item. | Choose an item. |  |
| Provide culturally and linguistically appropriate screening care. | Choose an item. | Choose an item. | Choose an item. | Choose an item. |  |
| Have a list of community screening/vaccination resources (e.g. free/reduced cost options, alternative clinic hours, various service locations). | Choose an item. | Choose an item. | Choose an item. | Choose an item. |  |
