# Supplementary material for: Improving the efficiency of integrated cancer screening delivery across multiple cancers: case studies from Idaho, Rhode Island, and Nebraska
Source: Implement Sci Commun. 2022 Dec 16;3:133. doi: 10.1186/s43058-022-00381-4 (PMC9756516; doi:10.1186/s43058-022-00381-4)
Supplement: Supplementary file 2 — Additional file 2: Supplemental Appendix 2. Facilitators and barriers to integrating policies, interventions, and strategies across cancer screening programs identified from informant interviews. [file 43058_2022_381_MOESM2_ESM.docx]

Supplemental Appendix 2 Facilitators and barriers to integrating policies, interventions, and strategies across cancer screening programs identified from informant interviews

| Facilitators | Barriers |
| --- | --- |
| Idaho Department of Health & Welfare (n=1) |  |
| Pre-implementation checklist to determine which interventions are feasible to implement to promote screening for multiple cancers | Limited staff time to implement interventions to promote cancer screening |
| Administrative support from health system managers to encourage staff buy-in | Staff turnover/staff shortages lead to less capacity in integrated intervention implementation |
| Buy-in from providers and staff to create unity around mission | Limited resources to implement interventions. For example, electronic medical record data enhancements are likely required for integrated delivery across multiple cancer screenings. |
| WellOne Primary Medical and Dental Care, Rhode Island (n=1) | |
| Provider referral encourages patients to be screened | Too much being asked of patients at once and patients can become overwhelmed |
| Being able to discuss as many preventive tests as possible at one time increases efficiency of navigator | Patients unclear of what is being asked and reluctant to be screened |
| Nebraska Department of Health and Human Services (n=1) | |
| Integration of services under one contract provides ease of administration | Possibility of local health departments focusing on only one service instead of all |
| Streamlining of contracts, processes, and resources creates efficiencies | Funding streams with unaligned start/end dates creates budget challenges |
